# Association of Walking Energetics With Amyloid Status: Findings From the Baltimore Longitudinal Study of Aging

**DOI:** 10.1093/geroni/igab046.1433

**Published:** 2021-12-17

**Authors:** Ryan Dougherty, Fangyu Li, Amal Wanigatunga, Qu Tian, Eleanor Simonsick, Murat Bilgel, Jennifer Schrack

**Affiliations:** 1 Johns Hopkins Bloomberg School of Public Health, Baltimore, Maryland, United States; 2 National Institute on Aging, Baltimore, Maryland, United States; 3 National Instute on Aging/NIH, Baltimore, Maryland, United States

## Abstract

Higher energetic costs for mobility are associated with slow and declining gait speed. Slow gait is linked to cognitive decline and Alzheimer’s disease (AD), but the physiological underpinnings are note well-understood. We investigated the cross-sectional association between the energetic cost of walking and amyloid status (+/-) in 174 cognitively unimpaired men and women (52%) aged 78.5±8.6 years. The energetic cost of walking was assessed as the average oxygen consumption (VO2) during 2.5 minutes of customary-paced overground walking. Amyloid status was determined from 11C-Pittsburgh compound B (PiB) positron emission tomography (PET) imaging. Average energetic cost of walking was .169±.0379 ml/kg/m and 30% of the sample was PiB+. In logistic regression adjusted for demographics, APOE-e4, body composition and comorbidities, each 0.01ml/kg/m higher energy cost was associated with 12% increased odds of being PiB+ (OR=1.12; 95% CI:1.01-1.24). Inefficient walking may be a clinically meaningful physiological indicator of emerging AD-related pathology.

